# Association between neck circumference and non-alcoholic fatty liver disease: cross-sectional analysis from ELSA-Brasil

**DOI:** 10.1590/1516-3180.2021.0095.R2.22062021

**Published:** 2022-01-17

**Authors:** Laura Luiza Menezes Santos, Maria de Fátima Haueisen Sander Diniz, Alessandra Carvalho Goulart, Sandhi Maria Barreto, Roberta Carvalho Figueiredo

**Affiliations:** I MSc. Dietitian, Universidade Federal de São João Del-Rei (UFSJ), Divinópolis (MG), Brazil.; II MD, PhD. Professor, Medical School and Clinical Hospital, Universidade Federal de Minas Gerais (UFMG), Belo Horizonte (MG), Brazil.; III MD, PhD. Clinical Epidemiologist and Researcher, Center of Clinical and Epidemiological Research, Hospital Universitário, Universidade de São Paulo (HU-USP), São Paulo (SP), Brazil.; IV MD, PhD. Professor, Medical School and Clinical Hospital, Universidade Federal de Minas Gerais (UFMG), Belo Horizonte (MG), Brazil.; V MD, PhD. Pharmacist and Professor, Universidade Federal de São João Del-Rei, Divinópolis (MG), Brazil.

**Keywords:** Liver diseases, Neck, Body composition, Adipose tissue, Weights and measures, Liver dysfunctions, Neck circumference, Body fat, Fatty tissue, Metabolic disorders

## Abstract

**BACKGROUND:**

Non-alcoholic fatty liver disease (NAFLD) has become a public health problem worldwide. Neck circumference (NC) is a simple anthropometric adiposity parameter that has been correlated with cardiometabolic disorders like NAFLD.

**OBJECTIVES:**

To investigate the association between NC and NAFLD, considering their obesity-modifying effect, among participants from the Longitudinal Study of Adult Health (ELSA-Brasil) baseline study.

**DESIGN AND SETTINGS:**

Cross-sectional study at the ELSA-Brasil centers of six public research institutions.

**METHODS:**

This analysis was conducted on 5,187 women and 4,270 men of mean age 51.8 (± 9.2) years. Anthropometric indexes (NC, waist circumference [WC] and body mass index [BMI]), biochemical and clinical parameters (diabetes, hypertension and dyslipidemia) and hepatic ultrasound were measured. The association between NC and NAFLD was estimated using multinomial logistic regression, considering potential confounding effects (age, WC, diabetes, hypertension and dyslipidemia). Effect modification was investigated by including the interaction term NC x BMI in the final model.

**RESULTS:**

The frequency of NAFLD and mean value of NC were 33.6% and 33.9 (± 2.5) cm in women, and 45.8% and 39.4 (± 2.8) cm in men, respectively. Even after all adjustments, larger NC was associated with a greater chance of moderate/severe NAFLD (1.16; 95% confidence interval [CI] for women; 1.05, 95% CI for men; P < 0.001). Presence of multiplicative interaction between NC and BMI (P < 0.001) was also observed.

**CONCLUSION:**

NC was positively associated with NAFLD in both sexes, regardless of traditional adiposity indexes such as BMI and WC. The magnitude of the association was more pronounced among women.

## INTRODUCTION

Non-alcoholic fatty liver disease (NAFLD) is characterized by a primary form of excessive fat accumulation in hepatocytes, which stands out because of its potential to evolve into inflammatory conditions, steatohepatitis, fibrosis, cirrhosis and hepatocellular carcinoma.^1–3^ Although diagnosed worldwide, it has variations in prevalence. In Brazil, its prevalence is estimated to reach about 20%-30%, with an increasing trend, such that it is becoming a public health problem.^4^

NAFLD is highly associated with excess weight, obesity, diabetes and dyslipidemia^5^ and more often affects men. On the other hand, premenopausal women are protected from developing NAFLD.^6^ In addition, NAFLD is a hepatic manifestation of metabolic syndrome, because it is associated with components such as insulin resistance (IR), abdominal fat, glucose intolerance and hypertension.^6^

The main methods for evaluating body fat and NAFLD are based on imaging. Particularly for NAFLD, ultrasound (USG) is considered to be a more accessible and reasonable diagnostic examination, compared with other imaging tests and with hepatic biopsy (the gold standard method).^7^ Transaminases tests, in turn, are not good diagnostic parameters because of their low specificity, since NAFLD does not always lead to liver enzyme abnormalities. These tests should therefore only be used as complements for another method.^6,8,9^ Both in epidemiological studies and in clinical practice, anthropometric indicators have been used as a proxy for NAFLD because they are more available, noninvasive and cost-effective than other existing methods used for assessing NAFLD.^10^

Neck circumference (NC) presents advantages over traditional anthropometric indices,^11^ given that this provides a separate estimate for the upper body subcutaneous adipose fat, which is considered to be just as pathogenic as visceral adipose fat. Presence of upper body adipose fat has been associated with several metabolic disorders,^12,13^ including NAFLD.^14–16^ Previous studies conducted among the Longitudinal Study of Adult Health (ELSA-Brasil) baseline participants already showed associations of NC with metabolic risk factors and with carotid intimal-media thickness, thus suggesting that NC measurement may be a potential marker for the risk of subclinical atherosclerosis and cardiovascular events.^17,18^ Additionally, it was concluded from a recent Brazilian study that NC can be used for screening of insulin resistance (IR) in patients with NAFLD.^19^ Some studies in other countries have demonstrated use of NC measurements as a predictor of NAFLD among obese and non-obese adult individuals, independent of other anthropometric indexes.^14–16,19,30^

## OBJECTIVE

The aim of the present study was to investigate the association between NC and NAFLD and whether this association is modified according to obesity status, among participants of the ELSA-Brasil baseline study.

## METHODS

### Design

This cross-sectional analysis used baseline data from ELSA-Brasil, a multicenter prospective cohort that enrolled 15,105 civil servants aged 35-75 years, from public institutions of higher education and/or research located in six Brazilian state capitals: Universidade Federal de Minas Gerais/ Centro Federal de Educação Tecnológica de Minas Gerais (UFMG-CEFET); Universidade de São Paulo (USP); Fundação Oswaldo Cruz (FIOCRUZ); Universidade Federal do Espírito Santo (UFES); Universidade Federal da Bahia (UFBA); and Universidade Federal do Rio Grande do Sul (UFRGS). The ELSA-Brasil baseline was established between 2008 and 2010 and further information about that study can be found in other publications.^21,22,23^ Approvals were obtained from the Research Ethics Committees of the institutions (CAAE 0016.1.198.000-06), and from the National Research Ethics Committee (CONEP 13065, Brasília, August 4, 2006). All the subjects signed an informed consent statement.

### Population study

Out of the total of 15,105 ELSA-Brasil baseline participants, we included in the present analysis all participants for whom complete data regarding the presence of non-alcoholic fatty liver through ultrasound USG and NC measurements were available. Participants for whom data on NC (n = 4) and liver ultrasound (n = 2,878) were missing, or for whom no images of reasonable/good or excellent quality were available, were excluded. We also excluded participants with the following characteristics: excessive alcohol intake (n = 712), self-reported history of cirrhosis and/or hepatitis (n = 989) and presence of thyroid dysfunction and/or use of medicine for thyroid dysfunction (n = 1065).

Excessive alcohol intake was assessed in terms of the types of drinks consumed, and their frequency and consumption patterns, and was classified as > 210 g of alcohol per week for men, and > 140 g per week for women. Cirrhosis and/or hepatitis was defined from self-reports of previous medical diagnosis and thyroid dysfunction, which was defined as changes in thyroid stimulating hormone (TSH) and T4 levels and/or levothyroxine or propylthiouracil use. The cutoff levels for hyperthyroidism were TSH < 0.4 mIU/l and free T4 > 1.9 ng/dl; and for hypothyroidism, TSH > 4.0 mIU/l and free T4 < 0.8 ng/dl.

In the end, 9,457 participants (5,187 women and 4,270 men) remained in the analytical sample of this study.

### Non-alcoholic fatty liver evaluation

Liver ultrasound examinations were performed by board-certified radiologists or by radiology technicians, after adequate training, using the same equipment: a high-resolution B-mode scanner (SSA-790A, Aplio XG, Toshiba Medical System, Tokyo, Japan) and a convex array transducer (model PVT-375BT, Toshiba Medical System, Tokyo, Japan), with a central frequency of 3.5 MHz and a fundamental frequency of 1.9-5.0 MHz. Subsequently, these B-mode hepatic ultrasound images were read by board-certified radiologists at the ELSA-São Paulo site, which was established as the ELSA-Brasil ultrasound reading center. The quality control protocol was verified by a senior ultrasound radiologist, by crosschecking the data. All examiners who performed ultrasound examinations were kept blind to the diagnosis of NAFLD.

The criterion for classifying hepatic attenuation of the ultrasound beam consisted of a standard B-mode ultrasound evaluation using a four-point visual classification system based on the degree of visualization of the diaphragm posterior to the right hepatic lobe.^24^ For the present study, the hepatic attenuation was classified as normal (complete viewing of the diaphragm), mild (partial, i.e. > 50% viewing of the diaphragm); or moderate/severe (< 50% viewing of the diaphragm or none). NAFLD was defined as mild or moderate/severe according to the above criterion.

### Anthropometric variables

NC was measured in centimeters (cm) using an inelastic tape, immediately above the cricoid cartilage and perpendicular to the long axis of the neck, with the participant in a sitting position and with head upright and eyes directed horizontally forward, in accordance with a standardized protocol.

Body mass index (BMI) was calculated by dividing the body weight measurement in kilograms by the squared height in meters (kg/m^2^). It was then classified as follows: normal (18.5 kg/m^2^ ≤ BMI < 25 kg/m²); overweight (BMI ≥ 25 and < 30 kg/m²); and obesity (BMI ≥ 30 kg/m²). Waist circumference (WC) was measured using standardized procedures and equipment and was used as a continuous variable (WHO, 1998). The participants were classified according to the obesity status presented, considering the BMI. All anthropometric measurements were performed by trained examiners.^25^

### Covariates

The demographic and socioeconomic characteristics that were evaluated were age, gender and education (elementary incomplete and complete, high school and college/university).

Presence of diabetes was defined as the presence of one of the following: medical history of diabetes; use of medication for diabetes treatment; fasting plasma glucose ≥ 126 mg/dl; two-hour glucose post load test ≥ 200 mg/dl; or HbA1C ≥ 6.5%. Presence of hypertension was defined as use of medication to treat hypertension, systolic blood pressure ≥ 140 mmHg or diastolic blood pressure ≥ 90 mmHg. Presence of dyslipidemia was defined as use of lipid-lowering agents or alterations in one of the following biochemical tests: cholesterol ≥ 200 mg/dl; triglycerides ≥ 150 mg/dl; high-density lipoprotein-cholesterol (HDL-C) < 50 mg/dl (women) and < 40 mg/dl (men); or low-density lipoprotein-cholesterol (LDL-C) ≥ 160 mg/dl.

### Statistical analysis

All analyses were stratified according to gender. Continuous variables were reported as the mean and standard deviation (± SD) and categorical variables as the frequency and percentage. The means for NC between the different degrees of NAFLD were compared by means of one-way analysis of variance (ANOVA). Multinomial logistic regression models were used to estimate the association between NC and NAFLD, considering the potential effects of the confounding variables. First, the crude odds ratios (ORs) were adjusted for age (continuous) (model 1); then adjusted for WC (continuous) (model 2); and subsequently adjusted for diabetes, hypertension and dyslipidemia (model 3). Lastly, a further adjustment was made through inclusion of the interaction term NC x BMI, which was inserted into the final model (model 4). The magnitude of the association was estimated using the OR and its respective 95% confidence interval (95% CI). All variables that presented associations in univariate analyses with a significance level lower than 0.20 were considered in the multivariable analysis, and only those that remained associated with the response variable at the P < 0.05 level were retained in the final model. All analyses were performed using the Stata 12.0 software (Stata Corporation, College Station, Texas, United States).

## RESULTS

Out of the 9,457 participants evaluated 5,187 were women and 4,270 were men, with a mean age of 51.8 ± 9.2 years. Among them, 33.6% of the women and 45.8% of the men presented some degree of NAFLD. The prevalence of a moderate/severe degree of NAFLD was 11.6% among the women and 20.3% among the men. In both sexes, the NC was larger at higher degrees of NAFLD. The main characteristics of our study participants are described according to their degree of NAFLD, for each gender, in Table 1.

**Table 1.** Main characteristics of the men and women participating in the study according to degree of non-alcoholic fatty liver disease (NAFLD). ELSA-Brasil, 2008-2010 (n = 9,457)

Variables	Women (n = 5,187)			Men (n = 4,270)		
	NAFLD			NAFLD		
	Normal	Mild	Moderate/ severe	Normal	Mild	Moderate/ severe
	**3,442 (66.4)**	**1,142 (22.0)**	**603 (11.6)**	**2,315 (54.2)**	**1,087 (25.5)**	**868 (20.3)**
**Age, years (SD)**	50.9 (8.9)	52.1 (8.6)	53.6 (8.5)	51.4 (9.7)	52.3 (9.2)	52.9 (8.9)
**Education, n (%)**						
Elementary incomplete	129 (3.8)	63 (5.5)	41 (6.8)	179 (7.7)	96 (8.8)	69 (8.0)
Elementary complete	164 (4.8)	87 (7.6)	42 (7.0)	206 (8.9)	101 (9.3)	62 (7.1)
High school	1278 (37.1)	459 (40.2)	250 (41.5)	790 (34.1)	362 (33.3)	290 (33.4)
College/university	1871 (54.4)	533 (46.7)	270 (44.8)	1140 (49.2)	528 (48.6)	464 (51.5)
**Diabetes, n (%)**						
No	3,061 (88.9)	910 (79.7)	370 (61.4)	1,962 (84.8)	810 (74.5)	506 (58.4)
Yes	380 (11.0)	232 (20.3)	233 (38.6)	352 (15.2)	277 (25.5)	361 (41.6)
**Hypertension, n (%)**						
No	2,599 (75.5)	705 (61.8)	305 (50.8)	1,573 (68.1)	628 (57.8)	414 (47.8)
Yes	841 (24.5)	436 (38.2)	296 (49.3)	740 (32.0)	458 (42.2)	452 (52.2)
**Dyslipidemia, n (%)**						
No	838 (24.4)	178 (15.6)	71 (11.8)	603 (26.1)	172 (15.8)	89 (10.3)
Yes	2,604 (75.7)	964 (84.4)	532 (88.2)	1,712 (73.9)	915 (84.2)	779 (89.8)
**Neck circumference, mean (SD)**	33.3 (2.2)	34.5 (2.5)	36.1 (2.7)	38.6 (2.6)	39.8 (2.7)	41.2 (2.8)
**BMI, n (%)**						
Normal	1,698 (49.3)	310 (27.2)	49 (8.1)	1112 (48.1)	267 (24.6)	80 (9.2)
Overweight	1,236 (35.9)	470 (41.2)	176 (29.2)	978 (42.3)	573 (52.7)	385 (44.3)
Obesity	508 (14.8)	362 (31.7)	377 (62.6)	224 (9.7)	247 (22.7)	403 (46,4)
**Waist circumference, mean (SD)**	83.9 (10.9)	90.7 (12.0)	99.4 (11.8)	90.6 (10.2)	96.9 (10.1)	104.4 (11.2)

n (%) or mean (standard deviation, SD); BMI = body mass index; ELSA= Estudo Longitudinal da Saúde do Adulto – Brasil.

Table 2 shows the crude and adjusted OR of NC in relation to NAFLD in the logistic models. NC was associated with NAFLD with a higher OR in relation to a mild degree of NAFLD (OR = 1.23, 95% CI: 1.19-1.27 for women; OR = 1.20, 95% CI: 1.17-1.24 for men) and a moderate/severe degree of NAFLD (OR = 1.56, 95% CI: 1.50-1.62 for women; OR = 1.45, 95% CI: 1.40-1.50 for men), compared with the reference category. Additionally, inclusion of WC in model 2 significantly altered the results.

**Table 2.** Crude and adjusted odds ratio between neck circumference (NC) and non-alcoholic fatty liver disease (NAFLD) for women and men, ELSA-Brasil, 2008-2010

	NAFLD	Model 0	Model 1	Model 2	Model 3	Model 4
**Women**	**Normal**	1.00	1.00	1.00	1.00	1.00
	**Mild**					
	NC	1.23 (1.19-1.27)**	1.22 (1.19-1.26)**	1.06 (1.02-1.10)*	1.04 (1.00-1.09)*	0.99 (0.95-1.05)
	WC	–	–	1.04 (1.04-1.05)**	1.04 (1.03-1.05)**	1.02 (1.01-1.04)**
	NC x BMI	–	–	–	–	1.00 (1.00-1.00)
	**Moderate/severe**					
	NC	1.56 (1.50-1.62)**	1.55 (1.50-1.62)**	1.20 (1.14-1.26)**	1.16 (1.10-1.23)**	1.06 (1.00-1.14)*
	WC			1.08 (1.07-1.09)**	1.08 (1.06-1.09)**	1.04 (1.03-1.06)**
	NC x BMI	–	–	–	–	1.00 (1.00-1.00)
**Men**	**Normal**	1.00	1.00	1.00	1.00	1.00
	**Mild**					
	NC	1.20 (1.17-1.24)**	1.20 (1.17-1.24)**	1.01 (0.97-1.05)	1.01 (0.97-1.05)	0.93 (0.88-0.99)
	WC			1.06 (1.05-1.07)**	1.06 (1.05-1.07)**	1.04 (1.02-1.05)**
	NC x BMI	–	–	–	–	1.00 (1.00-1.00)
	**Moderate/severe**					
	NC	1.45 (1.40-150)**	1.45 (1.40-1.50)**	1.06 (1.01-1.10)*	1.05 (1.00-1.10)*	0.95 (0.89-1.02)
	WC			1.12 (1.11-1.14)**	1.12 (1.10-1.13)**	1.09 (1.07-1.11)**
	NC x BMI	–	–	–	–	1.00 (1.00-1.00)

Reference for NAFLD = normal; *P < 0.05; **P < 0.001. Model 0: univariate; Model 1: adjusted for age; Model 2: model 1 + WC (waist circumference); Model 3: model 2 + diabetes, hypertension and dyslipidemia; Model 4: model 3 + interaction term NC x BMI (body mass index).

Among women, the magnitude of the association between NC and mild NAFLD (OR = 1.06; 95% CI: 1.02-1.10; P < 0.001) and moderate/severe NAFLD (OR = 1.20; 95% CI: 1.14-1.26; P < 0.001) NAFLD was significantly reduced after adjustment for WC. Among men, after adjustment for WC, the association of NC remained statistically significant only in relation to moderate/severe NAFLD (OR = 1.06; 95% CI: 1.01-1.10; P < 0.05). Adjustments for diabetes, hypertension and dyslipidemia did not modify the results found, in relation to either of the sexes.

To evaluate the effect modification due to obesity status (according to BMI) on the association between NC and NAFLD, the presence of multiplicative interaction between NC and BMI (P < 0.001) among women and men was also investigated. However, these analyses stratified according to normal weight (≥ 18.5 and < 25 kg/m^2^), overweight (25 ≤ BMI < 30 kg/m^2^) and obesity (> 30 kg/m^2^), and with adjustment for all confounding factors, including WC, only showed that NC was associated with a higher OR for having NAFLD (moderate/severe degree) among women with obesity. No such finding was observed among men (Tables 3 and 4).

**Table 3.** Crude and adjusted odds ratio between neck circumference (NC) and non-alcoholic fatty liver disease (NAFLD), stratified according to BMI (normal, overweight and obesity) among women, ELSA-Brasil, 2008-2010

	WOMEN				
	NAFLD	Model 0	Model 1	Model 2	Model 3
**Normal Weight**	**Normal**	1.00	1.00	1.00	1.00
	**Mild**				
	NC	1.09 (1.01-1.17)*	1.08 (1.00-1.16)*	0.99 (0.91-1.07)	0.98 (0.90-1.07)
	WC	–	–	1.05 (1.03-1.08)**	1.04 (1.02-1.06)*
	**Moderate/Severe**				
	NC	1.19 (1.01-1.41)*	1.18 (0.99-1.39)	0.99 (0.82-1.21)	0.99 (1.82-1.20)
	WC	–	–	1.09 (1.04-1.15)**	1.08 (1.03-1.15)*
**Overweight**	**Normal**	1.00	1.00	1.00	1.00
	**Mild**				
	NC	1.15 (1.08-1.22)**	1.14 (1.08-1.21)**	1.08 (1.02-1.16)*	1.07 (1.00-1.14)*
	WC	–	–	1.04 (1.02-1.06)**	1.04 (1.02-1.06)**
	**Moderate/Severe**				
	NC	1.24 (1.13-1.35)**	1.23 (1.12-1.34)**	1.10 (1.00-1.21)*	1.06 (0.96-1.17)
	WC	–	–	1.09 (1.06-1.13)**	1.08 (1.05-1.11)**
**Obesity**	**Normal**	1.00	1.00	1.00	1.00
	**Mild**				
	NC	1.11 (1.04-1.18)**	1.11 (1.04-1.18)**	1.07 (0.99-1.14)	1.05 (0.98-1.13)
	WC	–	–	1.02 (1.00-1.04)*	1.02 (1.00-1.04)*
	**Moderate/Severe**				
	NC	1.29 (1.22-1.38)**	1.29 (1.22-1.38)**	1.23 (1.15-1.32)**	1.20 (1.11-1.28)**
	WC	–	–	1.03 (1.01-1.05)*	1.03 (1.01-1.05)*

Reference for NAFLD = normal; *P < 0.05; **P < 0.001. Model 0: univariate; Model 1: adjusted for age; Model 2: model 1 + WC (waist circumference); Model 3: model 2 + diabetes, hypertension and dyslipidemia.

**Table 4.** Crude and adjusted odds ratio between neck circumference (NC) and non-alcoholic fatty liver disease (NAFLD), stratified according to BMI (normal, overweight and obesity) among men, ELSA-Brasil, 2008-2010

	MEN				
	NAFLD	Model 0	Model 1	Model 2	Model 3
**Normal Weight**	**Normal**	1.00	1.00	1.00	1.00
	**Mild**				
	NC	1.13 (1.05-1.22)**	1.13 (1.05-1.22)**	0.96 (0.88-1.05)	0.96 (0.88-1.05)
	WC	–	–	1.09 (1.07-1.13)**	1.09 (1.06-1.12)**
	**Moderate/Severe**				
	NC	1.43 (1.26-1.61)**	1.43 (1.26-1.61)**	1.09 (0.94-1.27)	1.11 (0.96-1.29)
	WC	–	–	1.19 (1.13-1.25)**	1.17 (1.11-1.23)*
**Overweight**	**Normal**	1.00	1.00	1.00	1.00
	**Mild**				
	NC	1.05 (0.99-1.10)	1.05 (0.99-1.10)	1.00 (0.95-1.07)	1.00 (0.94-1.06)
	WC	–	–	1.03 (1.01-1.06)*	1.03 (1.01-1.05)*
	**Moderate/Severe**				
	NC	1.18 (1.11-1.26)**	1.18 (1.11-1.26)**	1.05 (0.99-1.13)	1.03 (0.96-1.10)
	WC	–	–	1.11 (1.08-1.14)**	1.10 (1.07-1.13)**
**Obesity**	**Normal**	1.00	1.00	1.00	1.00
	**Mild**				
	NC	1.06 (0.99-1.14)	1.06 (0.99-1.14)	1.01 (0.93-1.10)	0.99 (0.92-1.09)
	WC	–	–	1.04 (1.01-1.07)*	1.04 (1.00-1.06)*
	**Moderate/Severe**				
	NC	1.14 (1.07-1.22)**	1.14 (1.07-1.22)**	1.03 (0.95-1.11)	1.03 (0.95-1.11)
	WC	–	–	1.07 (1.05-1.10)**	1.08 (1.05-1.10)**

Reference for NAFLD = normal; *P < 0.05; **P < 0.001. Model 0: univariate; Model 1: adjusted for age; Model 2: model 1 + WC (waist circumference); Model 3: model 2 + diabetes, hypertension and dyslipidemia.

## DISCUSSION

In this cross-sectional analysis on ELSA-Brasil participants, NC and NAFLD were independently associated, regardless of traditional anthropometric indexes and the presence of effect modification on the association due to obesity status, particularly among women. Our results support the hypothesis that obesity status (as assessed via BMI) modifies the association between NC and NAFLD in both sexes. However, after stratification according to BMI category, NC remained associated with a greater chance of moderate/severe NAFLD only among obese women, and not among men.

Recent studies on Asian populations also showed that NC was associated with NAFLD, in addition to the traditional indexes used (BMI and WC). One potential explanatory mechanism for the association between NC and NAFLD is that upper body subcutaneous fat, as estimated via NC, causes metabolic abnormalities such as accumulation of free fatty acids in the neck region. In addition, free fatty acids will generate a high amount of triglycerides to be stored in the liver, thus contributing to the accumulation of fat and to NAFLD.^15^ Another study showed that the upper region of the body, and more specifically the neck, is responsible for higher release of systemic free fatty acids than is the visceral region.^26^

NC measurement is feasible for use as an anthropometric method in investigations on metabolic disorders, given its harmlessness, low cost and lack of cultural restrictions. It therefore has good applicability in clinical practice and epidemiological studies with large samples, among other eternal measurements of body dimensions.^27^ Among these measurements, WC is frequently used and is known as a marker of abdominal fat, especially visceral adipose tissue, which makes this a measurement traditionally associated with cardiometabolic risks.^20,28,29^

However, WC has some important limitations that NC can highlight and overcome. One of these limitations is that the different anatomical parameters used for WC measurement (smallest circumference, umbilical scar, iliac crest, midpoint between the last rib and the iliac crest, among others) may significantly influence the measurement.^30^ Another is the variation that can occur in the abdominal region during the day, through with food intake, such that measurements are influenced by the time of day at which they are made.^30^ Moreover, it is not as practical and rapid to perform WC measurements in large population-based epidemiological studies. In particular, clothing is an obstacle during cold weather.^11^ In contrast, NC shows advantages because it is simple, easy and low-cost, has no intraindividual variation and is also considered to be a proxy measurement of upper body subcutaneous fat, since it is the only fat compartment that is separated and individualized without interference from other adipose tissues, and may be more easily measured.^13,16,30,31^

In addition, several studies have shown that NC measurements have positive associations with different cardiometabolic outcomes, such as type 2 diabetes,^32,33,34^ chronic kidney disease^35^ and metabolic syndrome.^36^ There is still a scarcity of data in the literature but, in agreement with our findings, three recent cross-sectional studies investigated the association between NC and NAFLD among Chinese adults with or without excess weight and suggested that NC measurement formed a marker for NAFLD.^14–16^ The findings of Hu et al. showed that NC was more effective for predicting NAFLD in women than in men, similar to our results.^14^ Huang et al. found a strong positive association in the highest NC quartile for the presence of NAFLD in both men and women, even after adjusting for age, BMI, WC and hip circumference (HC).^15^ Li et al. showed that among other anthropometric indices (BMI, WC and HC), NC tended to be higher with increased liver attenuation in both sexes.^16^ In addition, they concluded that although obesity is the major risk factor for the development of NAFLD (even a small increase in weight may increase the risk of fat in the liver), NC may be a useful and convenient indicator for detecting NAFLD in men with normal weight (BMI ≥ 18 kg/m^2^ and < 25 kg/m^2^), but not in women.

Regarding the differences in predicting moderate/severe NAFLD from NC measurements that were observed in our study between the sexes, the data in the literature are concordant with our findings. According to previous analyses from the Framingham Heart Study, which investigated the association of NC with cardiometabolic risk factors, fat deposits are more strongly associated with adverse risk factors among women than among men, because women store a higher proportion of free fatty acids in upper body subcutaneous adipose tissue, and especially because of the differences in the anatomical distribution of body fat that exists between the sexes.^13^ Women concentrate excess adipose tissue in the hip region (gynoid obesity, or “pear-shaped”); while men concentrate this in the central abdominal region (central obesity, or “apple-shaped”).^37^ Additionally, one recent study showed that although estrogen was a protective factor against NAFLD in women, it was not just men who were at increased risk of developing NAFLD. Significant age-related changes in NAFLD epidemiology among women may also potentially have physiopathological, clinical and therapeutic significance. For women for example, NAFLD epidemiology and physiopathology are modulated by the age at menarche and postmenopausal status. Those findings showed that, after the menopause, women displayed similar or even higher prevalence of NAFLD, compared with men, thus corroborating the effect of estrogens.^6,38^

In addition to finding that NC measurements were more effective for predicting the risk of NAFLD in women, Hu et al. concluded that these measurements might be more used among women in healthcare practices such as care and prevention of NAFLD.^14^ The findings from previous studies suggested that NC was a measurement that could contribute together with other anthropometric indices (BMI, WC and waist/hip ratio [WHR]). Thus, a synergistic effect towards better prediction of NAFLD, and possibly other risk factors for cardiovascular disease, was reported, due to the significant additive interaction that was found.^13,15^

Our study was the first to investigate and demonstrate the independent association of NC and NAFLD among Brazilian adults, and the results also support the hypothesis that obesity status modifies the association between NC and NAFLD. We found a statistically significant difference when the interaction term (NC x BMI) was added, among both men and women with moderate to severe NAFLD. Although different populations are compared in the literature, the synergistic effect also found in our study can be explained by differences in lifestyle, eating habits, distribution and body composition, as well as gender and race. In addition, the etiology of NAFLD also reflects complex interactions between genetic, neurohumoral, metabolic and stress-related factors.^9,26^

Moreover, the present study was carried out on a large sample of Brazilian adults, and strengthens the existing literature with data on the importance of measuring NC as a simple and easy predictor of NAFLD. However, it is important to highlight some limitations to the present study. First, the cross-sectional design does not allow inferences regarding causality in the association found. Second, the specificity and sensitivity of hepatic ultrasound for detecting NAFLD is variable. A study conducted by Goulart et al. using a subsample of ELSA-Brasil participants showed sensitivity of 85.1% and specificity of 73.4%, which may have impacted both the estimated prevalence of NAFLD and its association with NC.^24^ Lastly, the NAFLD data was not complete, because of exclusions of some hepatic ultrasound scans of poor quality. However, we do not believe that this loss was differential. Longitudinal analyses on ELSA-Brasil may contribute to better elucidation of the role of NC as a marker of cardiovascular risk.

## CONCLUSION

Our findings demonstrated that NC was significantly associated with NAFLD, after all adjustments in both sexes. We also observed the presence of multiplicative interaction between NC and BMI. These findings reinforced the positive association between NC and NAFLD, regardless of traditional adiposity indexes such as BMI and WC. Particularly among women, the magnitude of the association between NC and NAFLD was more pronounced.
